# Evaluation of X-ray Beam Collimation in Adult Chest Radiography

**DOI:** 10.7759/cureus.68489

**Published:** 2024-09-02

**Authors:** Khalid M Aloufi, Moawia Gameraddin, Ahmad S Albdrani, Mohammed M Alsani, Sultan A Alshoabi

**Affiliations:** 1 Diagnostic Radiology Technology, College of Applied Medical Sciences, Taibah University, Madinah, SAU

**Keywords:** image quality, radiation dose, area of interest, collimation, chest radiograph

## Abstract

Background: Restricting the irradiated volume can reduce X-ray scattering incidents on the image receptor. Proper X-ray collimation during medical imaging reduces a patient's dose while improving image quality. Even though the patient radiation dose due to chest X-ray imaging is low, the ‘as low as reasonably achievable’ (ALARA) principle should be satisfied, especially for young patients.

Purpose: To evaluate the accuracy of collimation in digital chest radiography.

Materials and Methods: Ninety-eight chest radiographs were studied retrospectively from February 2021 to December 2021. Chest images were collected from three main centers in the Madinah region of Saudi Arabia. The ratio of the field of interest area to the field of view (FOV) was measured and calculated to determine the accuracy of X-ray beam collimation.

Results: Out of 98 chest radiographs enrolled in the study, 87.8% (n=80) were adequately collimated, while 12.2% (n=18) were rejected due to inadequate collimation. The ratio of the field of interest collimated area of chest radiographs was 0.547, which indicated an acceptable value. Among the three centers, Center 2 showed higher, significant, adequate collimation than Center 1 (P<0.001) and Center 3 (P=0.007). There was a significant gender difference in collimation levels as the level of collimation of female chest radiographs is inferior to that of males (P>0.001).

Conclusions: The collimation of chest radiographs among the three centers was adequate. Based on the study findings, the X-ray beam collimation was sufficient, indicating good optimization and no unnecessary radiation exposure to patients and staff. The collimation of chest radiographs in females was significantly inferior to that of males.

## Introduction

Chest imaging using an X-ray beam is considered the most frequent radiation procedure used in medical imaging [1,2]. Various radiation modalities employ different types of radiation collimation. Nevertheless, the objective of collimation remains consistent as it entails restricting the X-ray beam to the precise region of concern, such as the chest, abdomen, or skull [3-5]. It minimizes the radiation field, thus reducing the radiation dose and obtaining optimum image quality [6]. However, reducing the patient dose should not affect the image quality [7,8]. 

Proper collimation can significantly reduce the patient dose by reducing the irradiated volume or avoiding repetition of the imaging procedure. Restricting the irradiated volume can reduce the number of X-ray scattering incidents on the image receptor (i.e., film cassette or digital receptors). This would decrease the patient radiation dose and improve the image quality [9]. The additional radiation doses received by the thyroid, eye lens, and abdominal organs can be reduced by applying accurate collimation [10,11]. While inadequate collimation can result in radiation exposure extending beyond the area of interest, additional radiation dose can be acquired from scattered radiation [12,13]. Moreover, the rejection of chest radiographic images due to improper collimation can be avoided. It has been found that 23.3% of the radiographic images involve improper collimation, positioning, motion artifacts, wrong labeling, exposure errors, detector errors, machine faults, re-requests from referring physicians, and picture archiving and communication system (PACS) issues [14].

According to the European Guidelines on Quality Criteria for Diagnostic Radiographic Images, the diagnostic requirements of chest posterior-anterior (PA) and lateral projections are characterized according to two main parts: image criteria and essential image details. Regarding the radiation dose from the chest PA and lateral projections, the entrance surface dose for a standard-size patient should not exceed 0.3 mGy and 1.5 mGy, respectively [15]. Although the patient radiation dose due to chest X-ray imaging is low, the ‘as low as reasonably achievable’ (ALARA) principle should be satisfied, especially for young patients [16-18]. This collimation can be performed manually or electronically [19]. Electronic collimation offers certain benefits, yet inherent risks are involved, such as potential unnoticed increases in patient dose and suboptimal image processing that could suppress diagnostic information [20,21]. There are also some advanced processing techniques in digital radiography, such as temporal subtraction, dual-energy subtraction, and computer-aided detection. The purpose of these is to reduce the impact of distracting anatomical background features and facilitate the identification of focused and minor abnormalities [22].

The European Commission's guidelines indicate accurate or adequate chest X-ray collimation criteria from the lung apices to the twelfth thoracic vertebra (T12). Furthermore, the collimation encompasses the soft tissue on the lateral sides of the chest, specifically at the level of the acromioclavicular joints, while the inferior collimation is situated at the level of the lower costal margin [23]. 

To our knowledge, few studies have investigated the accuracy of collimation used in digital chest radiography for adult patients in our region. This study aims to evaluate the accuracy of the collimation used in PA digital chest radiography for adult patients in the main centers of Madinah in Saudi Arabia, assess the X-ray beam collimation's impact on adult chest radiographs, examine patient gender influence on collimation, and compare the hospital centers' performance in collimation. This study differs from other studies in that it evaluates the chest collimation area in ratio rather than the collimated regions. The study is important for improving the principle of radiation protection in protecting patients, staff, personnel, and the public from unnecessary exposure to ionizing radiation.

## Materials and methods

Sample population

The study was conducted from February 2021 to December 2021. Data were collected from three main centers in the Madinah region for 98 patients (63 male and 35 female patients). Specific inclusion criteria were set for this study, including adult patients, PA projection, and accepted image quality. The exclusion criteria included pediatric patients, anteroposterior (AP) and lateral projections, and cut-off images.

The procedure of assessment of collimation and data collection

The chest radiographs were visually examined to determine the images that met the criteria of measurements. The X-ray machines utilized were a GE Healthcare/Siemens model AL01CII (Chicago, USA), a Siemens AXIOM Luminos dRF (Siemens Healthineers, Erlangen, Germany), and a GE Healthcare X-ray unit (Chicago, USA). The three X-ray machines were equipped with a high-frequency, three-phase, static digital unit with a maximum tube potential of 140 kVp and a maximum current of 600 mA. Additionally, there was a potter-bucky couch and an upright chest stand. The chest radiography approach was the same across all three centers. Every participant was examined in the erect position raising the cassette two inches above the shoulders and resting the chin on the cassette holder. They positioned their hands on the waist, dropped the shoulders, and centered to the level of the seventh thoracic vertebra. Exposure was made on deep suspended inspiration. The source image distance (SID) was 72 inches (183 cm), image receptor size was 35 × 43 cm (14 × 17 inches), lengthwise or crosswise. The exposure factors were mAs 4-8 mAs and 110-125 kV range.

All chest radiography images were visually examined to determine the images that met the inclusion and exclusion criteria. The inclusion criteria included chest images with good quality in terms of contrast and resolution and acceptable for diagnosis, while the exclusion criteria included degraded quality images. Two qualified radiologists determined the accepted images. It was found that 80 chest radiographs (87.8%) satisfied the inclusion criteria for this investigation, while 18 chest radiography images (12.2%) were excluded.

Measurements of collimated areas of the chest radiographs

**Figure 1:**
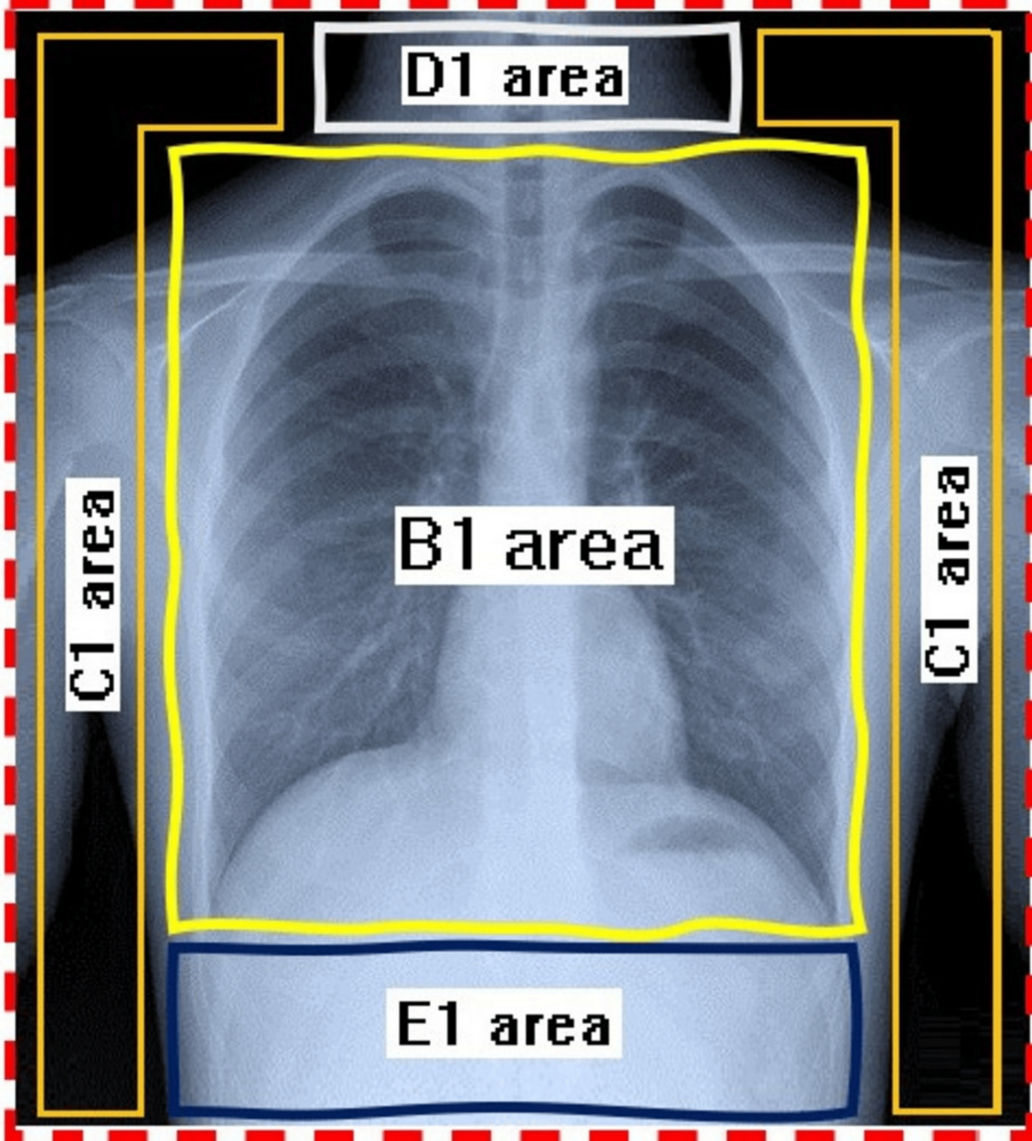
The determined collimated areas of a chest radiograph image: A1 (the red dashed line; field of view area), B1 (the yellow line; field of interest), C1 (the orange line; the margin area), D1 (the white line; the neck area), and E1 (the dark blue line; the abdominal area).

The collimated areas were measured and determined using Sante DICOM Viewer Lite (Santesoft Ltd., Athens, Greece). The image of each chest radiograph was divided into five areas, which were measured and assessed using the Sante DICOM Viewer Lite. The five areas were: the field of view (FOV) (A1) representing the radiographic area; the B1 area representing the field of interest; the field of lateral area (C1) representing the area from A1 to the lateral sides of the radiograph; the field of head and neck area (D1) representing the area from the B1 to the upper side of the radiograph; and the field of the abdominal area (E1) representing the area from B1 to the lower side of the radiograph, as shown in Figure 1.

The B1 area was calculated as 2 cm on all sides, according to the European Guidelines on Quality Criteria for Diagnostic Radiographic Images, which determines the collimation borders: inferior collimation - lower costal margin, superior collimation - shoulder joint, and lateral collimation - the level of the acromioclavicular joint. The accuracy of collimation is related directly to the value of the area of interest. Thus, if the ratio of the area of interest to the FOV area increases, the accuracy of collimation increases, whereas the accuracy of collimation decreases with any increase in the remaining three ratios (i.e., lateral, abdominal, and head and neck ratios). The increase in collimation can be attributed to the utilization of large image receptors that are commonly employed for imaging different body regions, ranging from the chest and abdomen to individual fingers [24].

Statistical analysis

The statistical calculations were performed using JASP software (JASP Team, 2019; jasp-stats.org). An independent sample student t-test was applied to investigate the influence of a patient's gender on the collimation accuracy. Also, one-way analysis of variance (ANOVA) and post hoc comparisons were applied to identify the performance of the hospital centers in chest radiography collimation. P-values lesser than 0.05 were considered significant.

## Results

A total of 98 cases of chest radiographs were selected for assessing the collimation. Eighty cases (87.8%) were adequately collimated, and 18 (12.2%) needed to be more adequately collimated. Table 1 summarizes the image evaluation of cases collected from the three centers.

**Table 1:** Image evaluation of cases collected from the three centers.

	Center 1	Center 2	Center 3	Total
Total cases	19	60	19	98
Gender (M/F)	9/10	40/20	14/5	63/35
Accepted (%)	14 (85)	52 (90)	14 (83.3)	80 (87.8)
Rejected (%)	5 (15)	8 (10)	5 (16.7)	18 (12.2)

The descriptive analysis of the examined areas is shown in Table 2.

**Table 2:** Descriptive analysis of the examined chest areas. A1, Field of view area; B1, Field of interest area; C1, Lateral area; D1, Head and neck area; E1, Abdominal area

	A1	B1	C1	D1	E1	
Mean	1368.405	737.294	346.191	28.015	256.906	
Std. deviation	257.446	163.989	173.752	19.136	116.668	
Minimum	819.218	457.822	52.449	0.019	25.091	
Maximum	1796.056	1324.468	722.679	84.663	540.132	

**Figure 2:**
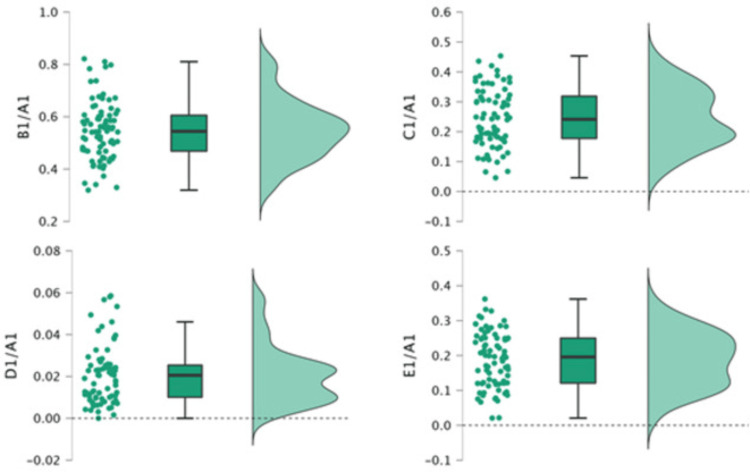
Raincloud plot of the areas examined. Ratios to the field of view area: B1/A1, Field of interest ratio; C1/A1, Lateral area ratio; D1/A1, Head and neck area ratio; E1/A1, Abdominal area ratio

The average (distributed) ratios of different FOV areas obtained from the three centers are shown in Table 3 and plotted in Figure 2. This figure shows the distributed FOV ratios for the different exposed areas (i.e., the field of interest, lateral, abdominal, and head and neck areas) collected from the three centers.

The average (distributed) ratios of different areas to the FOV area - the field of interest ratio (B1/A1), the lateral area ratio (C1/A1), the head and neck area ratio (D1/A1), and the abdominal area ratio (E1/A1) - were determined and calculated (Table 3). The distributed ratios of exposed areas of interest on the chest lateral and abdominal sides were 0.245 and 0.188, respectively. In contrast, the distributed ratio of exposed areas of interest in the head and neck area was 0.021. The maximum ratio was 0.547 for the field of interest (B1/A1), while the ratio of head and neck area (D1/A1) showed the minimum mean value (0.021). Thus, the exposed areas out of interest in the lateral and abdominal sides were 43.3% of the FOV area, and additional radiation dose here could be avoided by applying precise collimation. In contrast, the exposed areas out of interest in the head and neck area were 2.1% of the FOV area.

**Table 3:** Descriptive analysis of the ratios of the examined chest areas to the field of view area. Ratios to the field of view area: B1/A1, Field of interest ratio; C1/A1, Lateral area ratio; D1/A1, Head and neck area ratio; E1/A1, Abdominal area ratio

	Field of interest (B1/A1)	Lateral area (C1/A1)	Head and neck area (D1/A1)		Abdominal area (E1/A1)
Mean	0.547	0.245		0.021	0.188
Std. deviation	0.113	0.097		0.014	0.079
Minimum	0.319	0.046		0.000013	0.021
Maximum	0.822	0.454		0.059	0.362

**Figure 3:**
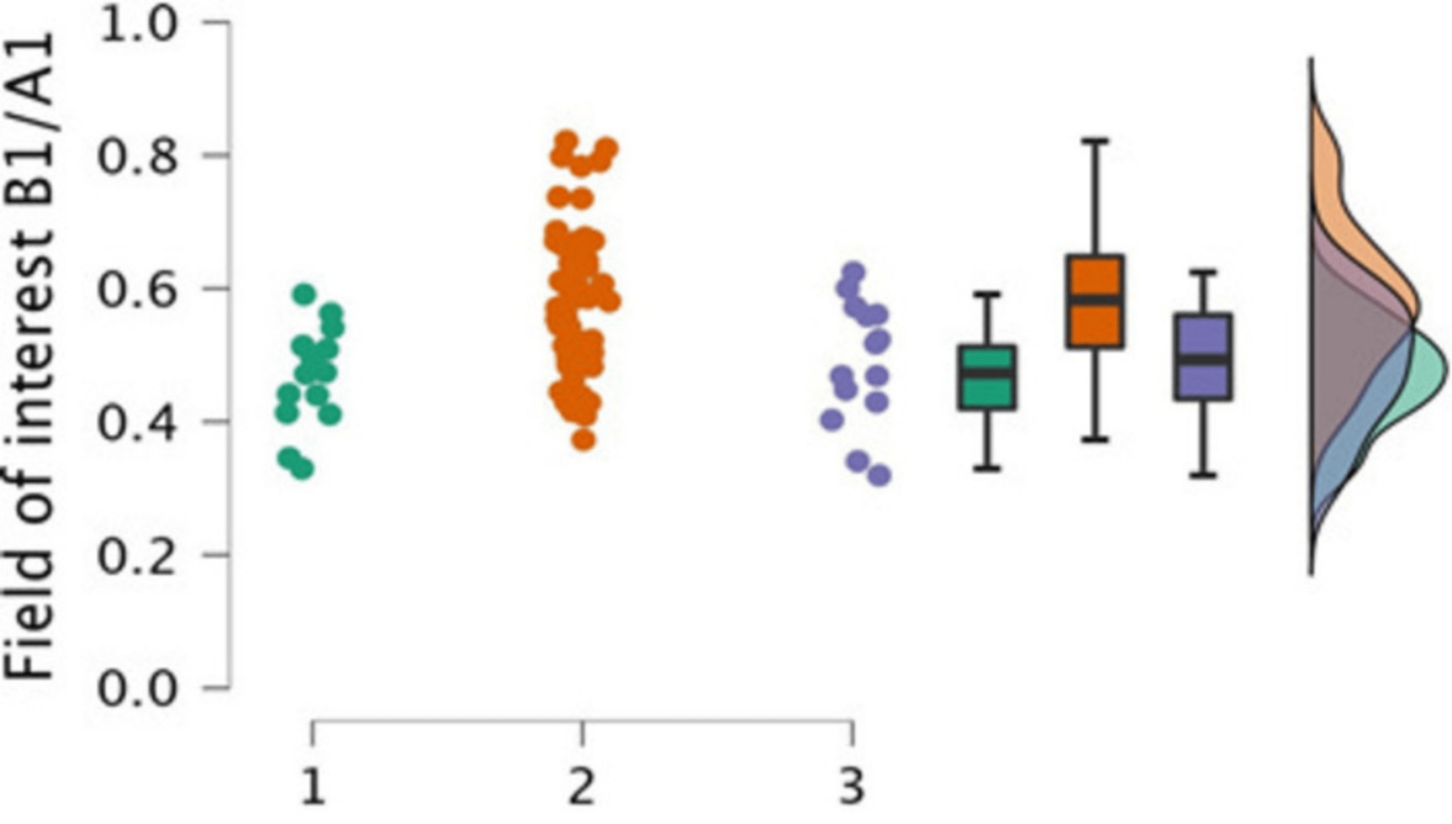
Raincloud plot of the field of interest area ratio to the field of view area (B1/A1) for the three centers. 1, Center 1; 2, Center 2; 3, Center 3

The ratios of the area of interest to the FOV area were compared for the three centers, as shown in Table 4, and then plotted in Figure 3. It was seen that there was a significant difference in the field of interest area to the FOV area ratio (B1/A1) for the three centers (P<0.001). Center 2 had the most significant FOV area ratio (B1/A1) compared to the other two centers.

**Table 4:** Descriptive analysis of the field of interest area to the field of view area ratio (B1/A1) for the three centers.

Centers	N	Ratio (B1/A1)		P-value
		Mean	SD	
Center 1	14	0.467	0.076	<0.001
Center 2	52	0.585	0.110	
Center 3	14	0.488	0.094	

The post hoc analysis for comparisons of the field of interest area ratios to the FOV area (B1/A1) among the centers showed that the accuracy of using collimation at Center 2 was significantly higher than that in Center 1 (P<0.001) and Center 3 (P=0.007), as shown in Table 5. Thus, the difference in accuracy shown in Table 5 for chest X-rays among the three centers was significant as P<0.001. In addition, post hoc analysis showed that the accuracy of using collimation at Center 2 was significantly higher than that in Center 1 (P<0.001) and Center 3 (P=0.007). This significant finding could be related to the notable variations in the experience levels of radiographers in these hospital centers.

**Table 5:** Post hoc comparisons of the field of interest area ratios to the field of view area (B1/A1) among the centers.

Centers	Mean diﬀerence	SE	T	ptukey	pbonf
Center 1 vs. Center 2	-0.117	0.031	-3.822	<0.001	<0.001
Center 1 vs. Center 3	-0.021	0.039	-0.545	0.850	1.000
Center 2 vs. Center 3	0.096	0.031	3.139	0.007	0.007

**Figure 4:**
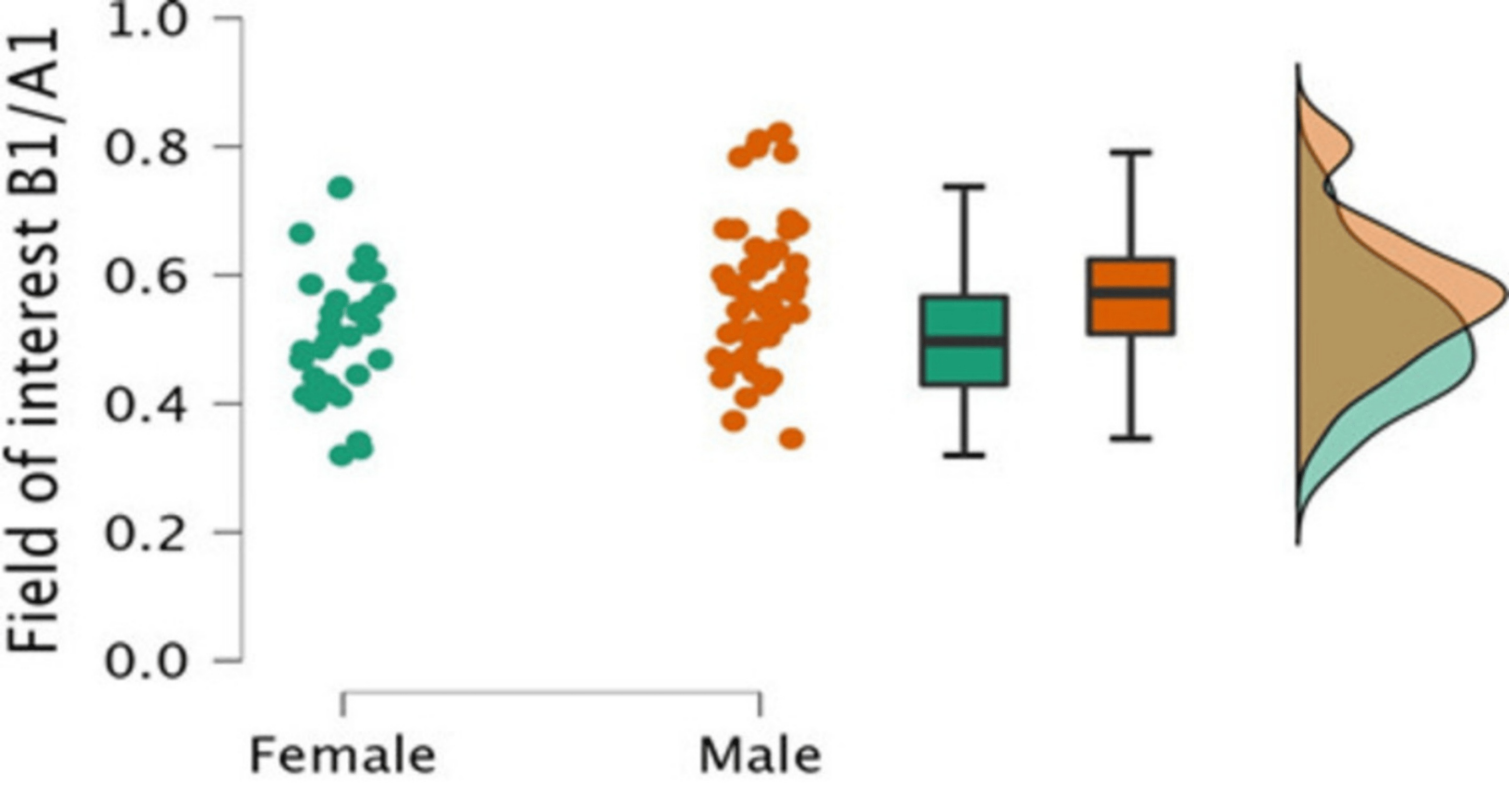
Raincloud plot of the field of interest area ratios to the field of view area (B1/A1) for male and female patients.

Moreover, the ratios of the area of interest to the FOV area were calculated regarding patient gender, as shown in Table 6, and then plotted in Figure 4. It revealed that the X-ray collimation accuracy in chest radiography for male patients was significantly higher than for female patients (P=0.007).

**Table 6:** Descriptive analysis of the field of interest area ratio to the field of view area (B1/A1) for male and female patients.

Interested area	Groups	N	Mean	SD	SE	P-value
Field of interest (B1/A1)	Female	31	0.505	0.106	0.019	0.007
	Male	49	0.574	0.110	0.016	

## Discussion

X-ray beam collimators are used to reduce the patient radiation exposure and the impact of secondary radiation on image contrast to maintain maximum diagnostic information with less discomfort to the patient. The current study found that the distributed ratio of the areas of interest was 0.547. A good agreement (90%) was found between this result and a result reported in the literature review [11]. 

It was determined in the study that the exposed areas of interest in the lateral, head and neck, and abdominal sides of chest radiographs were significant, which indicates improper collimation. The improper collimation might be attributed to electronic cropping, increased workload, and larger digital radiography plates. This finding is consistent with Nzotta et al., who reported that 78.6% of the chest radiographs were inadequately collimated and that additional radiation dose could be avoided by applying precise collimation [25]. Similarly, another study conducted by Okeji et al. on radiation exposure from diagnostic radiography, an assessment of X-ray beam collimation practice in some hospitals, found that inadequate beam collimation was present in 52% and 59%, respectively, of the radiographs evaluated in teaching hospitals and specialist hospitals. Additional radiation doses in these areas could be avoided by applying precise collimation [26]. 

The ratios of the area of interest to the FOV area were compared across the three centers. It was shown that there was a significant difference in the field of interest area to the FOV area ratio for the three centers. It was revealed that proper and accurate collimation at Center 2 was significantly higher than at Center 1 and Center 3, indicating adequate collimation. Consistent with this finding, Moi et al. assessed collimations used in chest radiographs for adult patients with the aim of radiation protection. Their study reported that 93.1% of the chest radiographs demonstrated adequate collimation [7]. In contrast, Ikamaise et al. reported that 95.2% of chest radiographs needed to be more adequately collimated [27]. The exposed areas that are of interest for general chest X-rays could contribute to unnecessary radiation doses, especially at the chest lateral and abdominal sides. The improper collimation could be attributed to the significant differences in the experience of radiographers that might exist in these centers.

The present study found a gender difference in collimation levels. It was observed that the effectiveness of implementing collimation for chest X-rays in female patients was lower than in males. In agreement with this finding, there was a gender difference in collimation levels reported by Adejoh et al. who showed that the collimation level at the second lumbar vertebra (L2) was more frequent in females than males [28]. This could be attributed to regional customs and more privacy provided to female patients, which may have led to inadequate patient positioning and poor X-ray collimation.

Radiographers should make considerable efforts to limit the primary beam to the area of diagnostic interest (ADI) to reduce patient exposure and increase image quality. This could be approached by arranging training courses, especially in radiation protection, to show the importance of X-ray collimation. Special consideration should be paid to the differences in the accuracy of collimation between centers to eliminate any unnecessary exposure to radiation of organs located outside the examined area. Though the average radiation dose from chest X-ray is small, the radiation protection principle ALARA should be applied.

It is important to consider certain limitations of this study while conducting future research. One of the limitations is the nature of the current study (retrospective), which needs more information on the patient position, exposure methods, exposure factors, collimation mode, and radiographic technologists' experience, necessitating future prospective studies to investigate collimator accuracy's impact on chest X-ray imaging. To ensure achieving these protocols, we suggest the following: (i) develop and implement standardized protocols for recording key variables across all imaging departments and research studies; (ii) utilize digital markers or sensors that can automatically track and record patient positioning and collimation accuracy during imaging procedures; and (iii) provide regular training for radiographic technologists on accurately recording these variables.

It is crucial to optimize the radiation dose while maintaining high image quality in diagnostic radiography. Therefore, it is recommended to address the correlation between radiation dosage and image quality, a topic that warrants more investigation in future studies.

## Conclusions

The assessment of the collimation of chest radiographs among the three centers was adequate. Based on this study's findings, X-ray beam collimation met the necessary standards in only one center (Center 2), indicating good optimization and no unnecessary radiation exposures to patients and staff. The collimation of chest radiographs in females was significantly inferior to that of males. Future work is required to select a larger population to assess the association of radiographers' experience and some technical factors in this interesting topic.
